# Supplementary material for: Knowledge, attitude and practice towards antenatal physical exercise among pregnant women in Ethiopia: A systematic review and meta-analysis
Source: PLoS One. 2023 Dec 14;18(12):e0295275. doi: 10.1371/journal.pone.0295275 (PMC10721098; doi:10.1371/journal.pone.0295275)
Supplement: S1 Table — (DOCX) [file pone.0295275.s002.docx]

**Newcastle Ottawa quality assessment of prevalence studies**

| **Included Studies** | **Assessment criteria** | | | | | | | |  |
| --- | --- | --- | --- | --- | --- | --- | --- | --- | --- |
|  | Representativeness of the sample | Sample size: | Non-respondents | Ascertainment of the exposure (risk factor): | The subjects in different outcome groups are comparable, based on the study design or analysis. Confounding factors are controlled | Assessment of the outcome: | Statistical test | total | Remark |
| Negash S,et al | 1 | 1 | 1 | 1 | 1 | 2 | 1 | 8 | good |
| Beyene MM et al | 1 | 1 | 1 | 2 | 1 | 2 | 1 | 9 | good |
| Sitot A et al | 1 | 1 | 1 | 1 | 1 | 2 | 1 | 8 | good |
| Janakiraman B et al | 1 | 1 | 1 | 2 | 1 | 2 | 1 | 9 | good |
| Beyene T et al | 1 | 1 | 1 | 1 | 1 | 2 | 1 | 8 | good |
| Gebregziabher D et al | 1 | 1 | 1 | 1 | 1 | 2 | 1 | 8 | good |
| Legesse M et al | 1 | 1 | 1 | 2 | 1 | 2 | 1 | 9 | good |
| Belachew DZ et al | 1 | 1 | 1 | 2 | 1 | 2 | 1 | 9 | good |
| Hailemariam TT et al | 1 | 1 | 1 | 2 | 1 | 2 | 1 | 9 | good |
| Ergicho SE et al | 1 | 1 | 1 | 2 | 1 | 2 | 1 | 9 | good |
| Bayisa D et al | 1 | 1 | 1 | 2 | 1 | 2 | 1 | 9 | good |
