# Supplementary material for: Knowledge, attitude and practice towards antenatal physical exercise among pregnant women in Ethiopia: A systematic review and meta-analysis
Source: PLoS One. 2023 Dec 14;18(12):e0295275. doi: 10.1371/journal.pone.0295275 (PMC10721098; doi:10.1371/journal.pone.0295275)
Supplement: S2 Table — (DOCX) [file pone.0295275.s003.docx]

**GRADE certainty of evidence**

|  | **Domains that lower certainty of evidence** | | | | | | **Factors that increase certainty of evidence** | | | **Overall quality** |
| --- | --- | --- | --- | --- | --- | --- | --- | --- | --- | --- |
| No of studies | Study design | Risk of bias | Inconsistency( heterogeneity) | Indirectness | Imprecision | Publication bias, | Magnitude of effect | Dose response gradient | Effect of confounding variables |  |
| 11 | Observational studies (evidence of certainty assessment started at low because of the design) | Serious (few number of studies included, the method employed in the study is cross-sectional, prone to bias) | Serious (significant heterogeneity detected) | Not serious (all studies the outcome variable objectively) | Not serious (all included studies have good sample size, narrow confidence interval of the estimate) | Not serious (No evidence of publication bias based on funnel plot and egger’s test) | Large magnitude of effect is observed in this meta-analysis | Not applicable for observational studies | All included studies have controlled the effect of confounding | low |
