# Supplementary material for: Knowledge, attitude and practice towards antenatal physical exercise among pregnant women in Ethiopia: A systematic review and meta-analysis
Source: PLoS One. 2023 Dec 14;18(12):e0295275. doi: 10.1371/journal.pone.0295275 (PMC10721098; doi:10.1371/journal.pone.0295275)
Supplement: S1 File — (DOCX) [file pone.0295275.s004.docx]

**Pubmed**

(((((((((knowledge[Title/Abstract]) OR (attitude[Title/Abstract])) OR (practice[Title/Abstract])) AND (antenatal physical exercise[MeSH Terms])) OR (physical activity[MeSH Terms])) AND (pregnant women[MeSH Terms])) OR (associated factors[Title/Abstract])) OR (determinants[Title/Abstract])) OR (predictors[Title/Abstract])) AND (Ethiopia[Title/Abstract]) ((((((((knowledge [Title/Abstract]) OR (attitude[Title/Abstract])) OR (practice[Title/Abstract])) AND (antenatal physical exercise[MeSH Terms])) AND (pregnant women[Title/Abstract])) OR (associated factors[Title/Abstract])) OR (predictors[Title/Abstract])) OR (determinants[Title/Abstract])) AND (Ethiopia[Title/Abstract])

**Google scholar**

All in title: knowledge, attitude, practice of antenatal physical exercise among pregnant women AND associated factors in Ethiopia. All in title: "antenatal physical exercise" AND "pregnant women" Ethiopia All in title: "pregnant women’ knowledge on antenatal physical exercise” AND Ethiopia All in title: "antenatal physical activity" AND Ethiopia All in title: “physical activity" AND "pregnant women’s attitude" All in title: "pregnant women’s practice on antenatal physical exercise" AND Ethiopia

**African Journals online**

“Knowledge, attitude and practice of pregnant women towards antenatal physical exercise and its associated factors”

“Determinants of antenatal physical exercise among pregnant women in Ethiopia”

**Web of science**

(((((((((((antenatal physical exercise) OR (antenatal physical activity [all fields])) OR (exercise])) AND (knowledge[topic])) OR (practice[all fields])) AND (pregnant women[all fields])) OR (associated factors [topic])) OR (determinants[all fields])) OR (predictors[topic]))) AND (Ethiopia[title])) OR (all fields])
