# Supplementary material for: Knowledge, attitude and practice towards antenatal physical exercise among pregnant women in Ethiopia: A systematic review and meta-analysis
Source: PLoS One. 2023 Dec 14;18(12):e0295275. doi: 10.1371/journal.pone.0295275 (PMC10721098; doi:10.1371/journal.pone.0295275)
Supplement: Attachment — Submitted filename: Response to the editor and reviewers.docx [file pone.0295275.s005.docx]

**Response to the editor and reviewers**

On behalf of all authors of the submitted manuscript, I would like to express my gratitude for your constructive comments to further enrich our work. We have made a thorough revision in accordance with the given comments. Kindly find the below mentioned point by point response/reflections or actions taken to forwarded comments and suggestions.

R: Please ensure that your manuscript meets PLOS ONE's style requirements, including those for file naming.

A: we have revised the manuscript in accordance with PLOS One’s style requirements. Figures and supporting files name have changed to harmonize it with the journal’s requirement.

R: We noticed you have some minor occurrence of overlapping text with the following previous publication(s), which needs to be addressed:

A: Thank you, we have synthesized and rephrased some overlapping texts to make our content unique. References are corrected and appropriate references that were missed on the previous version are cited on the revised version.

R: Please include captions for your Supporting Information files at the end of your manuscript, and update any in-text citations to match accordingly. Please see our Supporting Information guidelines for more information: <http://journals.plos.org/plosone/s/supporting-information>.

A: We have included captions for the supporting file on the revised version of the manuscript at the end of the manuscript under the heading “Supporting information”

R: Writing language needs to be improved

A: We have made revisions to make the manuscript readable

R: The objective stated in the abstract does not align with the objective stated at the end of the introduction and the title of the study. In the abstract, it mentions associated factors to awareness and so on, while the main objective is to determine the level.

A: we have modified the objective on the abstract section to make it consistent with the rest of the manuscript sections.

R: In the abstract, it is better to use "pooled level" or "pooled proportion" instead of "pooled prevalence" to accurately describe the findings.

A: modified accordingly

R: In the section regarding the databases searched, it is advisable to remove Google because the use of this database is not customary in scientific texts

A: correction taken

R: Quality assessment section should be written in detail with a description of the method, such as the number of assessors and how disagreements were resolved, etc

A: Thank you; we have elaborated this section a little bit. As it is already mentioned two authors were involved in the quality assessment activity and an author has involved when there was disagreement between the two assessors. The remark given by the third author was taken to declare the assessment result of the study in case of disagreement between the two assessors.

R: The first paragraph of the findings should be divided into two subheadings: search results and the characteristics of studies, and the explanations for each part should come under the same part.

A: Thanks, we have revised and presented the first paragraph of the result section in two sub-headings on the revised version.

R: Font plots related to the variables of knowledge, attitude, and practice will be designed in a single figure and labeled as 'Figure a' for knowledge, 'Figure b' for attitude, and 'Figure c' for practice. Additionally, sensitivity analysis plots will be generated.

A: Thank you, it is valid comment, but we couldn’t manage it in that way, we had tried to present the funnel plots of the three variables in single figure, however it becomes more congested and not easily readable. As a result we prefer to retain a standalone figures for each of the three variables.
