# Supplementary material for: Knowledge, attitude and practice towards antenatal physical exercise among pregnant women in Ethiopia: A systematic review and meta-analysis
Source: PLoS One. 2023 Dec 14;18(12):e0295275. doi: 10.1371/journal.pone.0295275 (PMC10721098; doi:10.1371/journal.pone.0295275)
Supplement: Attachment — Submitted filename: Response to reviewers.docx [file pone.0295275.s006.docx]

**Response to reviewers and the editor**

On behalf of all authors of the submitted manuscript, I would like to express my gratitude for your constructive comments to further enrich our work. We have made a thorough revision in accordance with the given comments. Kindly find the below mentioned point by point response/reflections or actions taken to forwarded comments and suggestions.

R: The manuscript contains formal errors of scientific writing that need to be corrected urgently.

A: thank you, We have made a thorough revision to abide with scientific writings

R: The Conclusions are speculative and should be rewritten with precision.

A: we have revised and tried to be more precise on the revised version.

R: The submission of a properly completed PRISMA checklist is mandatory for the next submission.

Correcting the manuscript according to the PRISMA checklist (i.e. providing all the information requested and in the order indicated) is mandatory

A: we have rechecked the PRISMA checklist and we have revised the manuscript in accordance with the check list

R: You presented your reports via present tense, please use “past tense “ instead. This is a basic principle of reporting manuscripts.

A: thank you, we have revised it accordingly

R: Where P.value=0.00000…, write it as P<0.001 (please, fix this issue across the manuscript)

A: corrected accordingly

R: Please be consistent in using words, phrases, and statistical values across your manuscript

A: Thank you, we have corrected the inconsistency in terminologies that we have identified

R: Please avoid using the term “ predictors ”! use “associated factor” instead. Because this epidemiological term is employed when studies conducted using strong models ( i.e cohort, follow-up et ) are considered. I found only one article included your synthesis conducted using a cohort study design ( refer to Table 1)

A: we replaced the word predictors with associated factors

R: Meta-analysis should be spelled as meta-analysis, - Add “A” to the subtitle ….(correct ) A systematic review and meta-analysis

A: Thank you, modified accordingly

R: Abstract, A total of eleven studies….. Insead start with, A total of 11 studies were…….. . . NB: We should mention the absolute if frequencies are less than 10 and otherwise text if less than 10.

Conclusion: ……desirable level??…… , replace with “ the recommended level”.

….. are significant predictors of antenatal physical exercise practice. Replace with…were found to be associated with a good level of practice toward antenatal physical exercise.

A: Thank you, all the suggestions are relevant, we took corrections on the revised version on the aforementioned comments

R: Accordingly there is no registered ……replace “is” with “was”

A: Correction taken

R: Last line …..Include PRISMA statement as supplementary /additional file 1

A: we have included a linking statement to additional file 1.

R: Search strategies and source information, Please cite ….CoCo/PEO guidelines???

A: appropriate references is cited on the revised version

R: - I did not find search detail in the supporting file and also you need to cite in the text

A: search terms employed in each database is uploaded as a supplementary file,

R: You considered unpublished articles/preprints for your study while they are not reputable sources of evidence as they are not peer reviewed and hence are not recommended to be included! Can you explain/resolve this issue?

A: though it is obvious that peer reviewed articles tend to have high quality evidence, it doesn’t mean that unpublished evidences are with poor quality. As long as I know both published and unpublished articles can be included in a systematic review and meta-analysis as far as they fulfill the eligibility criteria and quality assessment parameters employed in the method section.

R: Is there any justification why you prefer to include articles published at any time until the end of our search (July12, 2023)? As this is a protracted/long period. There might be new policies, recommendations, and practices during these long periods

A: in principle it is true that changes in policy and recommendations could affect personal behaviors like antenatal physical activity. However, in our case scientific report regarding antenatal physical exercise is a recent development. All the included studies are between 2015-2023; hence we have no reason to limit our search time.

Data processing and analysis

R- Please cite.. STATA software version 17 for analysis(???).NB, software, guidelines, protocols, etc need to be cited

A: reference cited on the revised version

R- What is ………pooled level of good vaccine cold chain management practice in Ethiopia???. Please remove it! I understand that you wrote the manuscript on the background of one of you’re your previous works.

A: Thank you, apologies for such inadvertent errors; you have well understood the context in which the error has occurred, thanks indeed. I have corrected it to the concept under inquiry.

R: Please cite articles for each of these! The included studies are three in Tigray region (citation), while Amhara region (citation ), SNNPR region (citation), and Addis Ababa city (citation), contributed two studies each(citation), and Harar (citation), and Sidama regions (citation), represented by one study each. All of the included articles are cross-sectional studies (citation), and the included sample ranges from 240 to 806 pregnant women

A; Thank you, references are cited on the revised version

R- Table 1 row 1 : should it be study year or publication year?

A; both are applicable, for published articles we took publication year whereas we can only take study year for unpublished sources. If it has to be included we can modify to (study/publication year)

R: Knowledge of pregnant women towards antenatal physical exercise

- ….....please re-write 46.04% with 95% CI (44.45%-47.63%) as ……was 46.04% (95% CI: 44.45,4.63) . would you do similar across the manuscript?

A: Corrected accordingly

R; • From the sociocultural standpoint, women's awareness, and perceptions about the domain under consideration are different among people living in a country. How do authors justify pooling the estimate of good knowledge, favorable attitude, and good practices as the studies are completely heterogeneous? Can you mention this as a limitation as it could be a source of concern for the use of the results?

A: One of the reasons for having a systematic review and meta-analysis is the existence of inconsistent level of women’s KAP towards antenatal physical exercise. Pooling will not be a problem as far as the measurements of variables are consistent. If the level of awareness is unequivocal across different settings, there will not be a rationale to compute a single estimate using a meta-analysis.

• You used an article published in the English language only, please add to the limitation of the study

A: thank you, incorporated as a limitation on the revised version of the manuscript

R: Included studies were included from a few regions of your country. What do you comment on about the representativeness of the information at the national level?

A: The included studies are few and might not fully represent all administrative regions in Ethiopia; it would have been more informative if there were additional studies to be included in this review and meta-analysis. It has already mentioned as a limitation to enable readers made careful interpretation of the results.

R: Add implications for research and policy

A: I have included its implication for policy and research on conclusion part of the revised version of the manuscript.

R: Please arrange key words in alphabetical orders

A: Thank you, it is corrected on the revised version

R: On subgroup analysis paragraph two please correct this number 4949.99%

A: thank you, the typos are corrected

R: On discussion part level of good knowledge comparison with different country please write level of knowledge in each country and briefly clarify justification for the difference in level of knowledge with scientific reason

A: we have made comparison across different settings, and actual and speculative justifications are forwarded for the observed differences on the variable under inquiry.
